# Exploring U.S. Shifts in Anti-Asian Sentiment with the Emergence of COVID-19

**DOI:** 10.3390/ijerph17197032

**Published:** 2020-09-25

**Authors:** Thu T. Nguyen, Shaniece Criss, Pallavi Dwivedi, Dina Huang, Jessica Keralis, Erica Hsu, Lynn Phan, Leah H. Nguyen, Isha Yardi, M. Maria Glymour, Amani M. Allen, David H. Chae, Gilbert C. Gee, Quynh C. Nguyen

**Affiliations:** 1 Department of Family and Community Medicine, University of California San Francisco, San Francisco, CA 94110, USA; 2 Department of Health Sciences, Furman University, Greenville, SC 29613, USA; shaniece.criss@furman.edu; 3 Department of Epidemiology & Biostatistics, University of Maryland School of Public Health, College Park, MD 20742, USA; pdwived1@umd.edu (P.D.); dinahuang26@gmail.com (D.H.); jkeralis@umd.edu (J.K.); qtnguyen@umd.edu (Q.C.N.); 4 Department of Public Health Science, University of Maryland, College Park, MD 20742, USA; ehsu@terpmail.umd.edu (E.H.); lphan04@terpmail.umd.edu (L.P.); leahn98@umd.edu (L.H.N.); iyardi@terpmail.umd.edu (I.Y.); 5 Department of Epidemiology & Biostatistics, University of California San Francisco, San Francisco, CA 94158, USA; maria.glymour@ucsf.edu; 6 Divisions of Community Health Sciences and Epidemiology, University of California, Berkeley, CA 94704, USA; amaniallen@berkeley.edu; 7 Department of Global Community Health and Behavioral Sciences, Tulane School of Public Health and Tropical Medicine, New Orleans, LA 70112, USA; dchae@tulane.edu; 8 Department of Community Health Sciences, University of California, Los Angeles, CA 90095, USA; gilgee@ucla.edu

**Keywords:** social media, minority groups, racial bias, big data, content analysis

## Abstract

**Background:** Anecdotal reports suggest a rise in anti-Asian racial attitudes and discrimination in response to COVID-19. Racism can have significant social, economic, and health impacts, but there has been little systematic investigation of increases in anti-Asian prejudice. **Methods:** We utilized Twitter’s Streaming Application Programming Interface (API) to collect 3,377,295 U.S. race-related tweets from November 2019–June 2020. Sentiment analysis was performed using support vector machine (SVM), a supervised machine learning model. Accuracy for identifying negative sentiments, comparing the machine learning model to manually labeled tweets was 91%. We investigated changes in racial sentiment before and following the emergence of COVID-19. **Results:** The proportion of negative tweets referencing Asians increased by 68.4% (from 9.79% in November to 16.49% in March). In contrast, the proportion of negative tweets referencing other racial/ethnic minorities (Blacks and Latinx) remained relatively stable during this time period, declining less than 1% for tweets referencing Blacks and increasing by 2% for tweets referencing Latinx. Common themes that emerged during the content analysis of a random subsample of 3300 tweets included: racism and blame (20%), anti-racism (20%), and daily life impact (27%). **Conclusion:** Social media data can be used to provide timely information to investigate shifts in area-level racial sentiment.

## 1. Introduction

Recent reports [1,2] have noted a rise in prejudicial attitudes and discrimination against Asian Americans in the United States in relation to COVID-19 [3]. For example 1843 incidents of discrimination and harassment against Asians were reported to Stop Asian American Pacific Islander (AAPI) Hate between 19 March 2020 and 13 May 2020 [4]. Beginning in February and March 2020, there was an increase in the use of stigmatizing language to refer to COVID-19 as the “Wuhan virus,” “Chinese virus,” and “Asian virus,” [5] leading COVID-19 to become increasingly associated with Asians. A rise in negative racial attitudes and racial bias can have significant social, economic, and mental and physical health impacts [6,7,8]. Prior research has shown that community-level racial climate is related to birth outcomes [9,10,11], cardiovascular outcomes [12], and mortality [13] of the area. Such attitudes reflect cultural racism, defined as the infusion of the ideology of racial inferiority in the values, language, imagery, symbols, and unstated assumptions of the larger society [14]. Cultural racism is displayed through media, stereotyping, and norms within society and its institutions. In this way, cultural racism is systemic and produces an environment where institutional and individual-level discrimination can thrive [14], both of which have been associated with adverse mental and physical health outcomes [6,15].

Prejudicial attitudes and speech are important aspects of cultural racism, but can be challenging to monitor. Prejudicial attitudes have been assessed via surveys [16], assessment of vignettes [17], and through performance-based tasks (e.g., implicit association tests) [18]. However, these methods are limited in several ways. For example, reporting in socially desirable ways is a common concern for surveys, particularly when assessing attitudes about socially sensitive topics, and can lead to response bias. Furthermore, these methods are employed in artificial settings. Surveys require participants to respond to forced-choice answers using predetermined questions. These items may not reflect the emergence of new forms of prejudicial language and may not capture the range of potential responses. In the present study, we examine data that have been collected in response to the emergence of COVID-19 through the popular online platform—Twitter.

Millions of tweets are sent daily by users across the globe, and 90% of Twitter users make their profile public [19]. Perceived anonymity associated with online spaces may decrease self-censorship of socially unacceptable views and increased willingness to express attitudes that are less likely to be reported in survey interviews due to social desirability response bias [20]. Leveraging data from social media may be one way to circumvent some of the limitations of traditional self-reported measures and help capture attitudes about sensitive topics such as racial prejudice and bias.

In addition, social media represents a rapid mode of communication and is a way to share frequent updates and information. Data derived from social media can therefore reveal aspects of people’s daily lives and rapidly fluctuating social and cultural processes at work both within and across the United States. The continuous and pervasive nature of social media use provides the opportunity to examine patterns of racial attitudes. This study aims to describe variation in racial sentiment based on the sentiment of tweets using race-related terms before and following the emergence of COVID-19 in the United States and describe themes of Twitter discussions related to race and COVID-19 to more fully explore the context of community-level racial sentiment. 

## 2. Methods

### 2.1. Overview

We used a mixed-methods approach that integrated state-of-the art machine learning sentiment analyses of publicly available tweets alongside qualitative content analyses of a subset of those tweets. First, a random 1% sample of publicly available tweets was collected from November 2019 to June 2020, using Twitter’s Streaming Application Programming Interface (API). Details of the data collection process including the full keyword list have been previously published [21]. We restricted our analyses to English language tweets from the United States with latitude and longitude coordinates or other “place” attributes that permitted the identification of the state where the tweet was sent. All tweets for the sentiment analysis used one or more of 518 race-related keywords. These keywords were compiled from racial and ethnic categories used by prior studies examining race-related online conversations [22,23] and an online database of racial slurs [24]. Tweets were classified into four main racial/ethnic categories: Asians, Blacks, Latinx, and Whites according to the keywords used. The analytic sample included 3,377,295 tweets from 521,161 Twitter users. Sentiment analysis was performed on this data set to examine temporal trends in negative racial sentiment before and after the emergence of COVID-19. The qualitative content analysis included a subset of tweets (*n* = 3300) that used race-related terms and one or more of 75 coronavirus-related keywords from February to April 2020 when public awareness of COVID-19 became widespread in the United States. Coronavirus-related terms were compiled from the medical and scientific community [25] and from colloquial terms used online. This study was determined exempt by the University of California, San Francisco Institutional Review Board (18-24255).

### 2.2. Sentiment Analysis

Twitter data were cleaned and processed for the sentiment analysis. We dropped duplicate tweets according to their “tweet_id” and pre-processed the tweets to remove stop words, emojis, urls, punctuations, and hashtag signs. We utilized support vector machine (SVM), a supervised machine learning model to predict the sentiment of each tweet. Support vector machine (SVM) is used for text classification in natural language processing tasks [26]. Since SVM is a supervised machine learning model, it needs training data, typically human-labeled data, to “learn” what people consider a negative or positive tweet. We obtained training data from manually labeled Sentiment140 (*n* = 498) [27], Kaggle (*n* = 7086) [28], Sanders (*n* = 5113) [29], and 6,481 tweets labeled by our research group. Sentiment140, Sanders, and Kaggle datasets are all publicly available training datasets specifically labelled for sentiment analysis. First, we labeled negative tweets (assigned a value of 1) to all other tweets–positive or neutral tweets (assigned value of 0). We used 5-fold cross validation to assess the model performance and reached a high level of accuracy for the negative classification (91%) and a high F1 score (84%). Tweets were also labeled as positive or not positive. We similarly used 5-fold cross validation and achieved an accuracy of 89% and a F1 score of 81%. Once the SVM models have been trained for negative and positive sentiment prediction, we then applied the SVM negative and positive sentiment model to label new tweets we collected from 2019–2020.

Statistical analyses were implemented with Stata MP16 (StataCorp LP, College Station, TX, USA). The prevalence of negative sentiment against time (time series) figures were generated using R (R Foundation for Statistical Computing, Vienna, Austria) [30]. LOESS (locally estimated scatterplot smoothing) was performed to fit smooth curves to the daily prevalence of negative sentiment.

### 2.3. Content Analysis 

While the sentiment analysis provides us with a classification of the sentiment of the tweets, the qualitative content analysis is used to provide complementary information on the topics and themes discussed in these tweets. To provide a more contextual-level understanding of social climate related to race and identify emerging themes of Twitter discussions related to race and COVID-19, we conducted a qualitative content analysis of a random subsample of COVID-19 and race-related tweets. Two members of the research team developed a codebook (i.e., a list of codes and definitions representing the emerging themes) based on a literature review and coding and discussing 200 tweets from the above sample. This consensus building process enhanced the codebook by clarifying operational definitions, thereby increasing internal validity. The final categories or emergent themes included racism, blame, anti-racism, misinformation, U.S. politics, news, call for action, and daily life impacted by COVID-19. Using this coding scheme, each of the two research members coded 100 tweets with two research assistants (400 tweets total) to test inter-rater agreement. Once Cohen’s kappa reached 70% or higher for each coder pair, indicating substantial agreement [31], all team members independently coded 2100 tweets. The researchers double-coded 1200 tweets throughout the process (800 tweets in the beginning and 400 in the last third of the sample). Any disagreements in coding were discussed until consensus was met. Kappa agreement of 70% or higher was met for each inter-coder session. 

## 3. Results

From November 2019 to June 2020, we collected 3,377,295 tweets containing at least one of the relevant keywords pertaining to a racial or ethnic group. From a list of 518 terms, 20 terms comprised 80% of all tweets that referenced a racial or ethnic minority group. The top race-related Twitter terms were: “n*gga/n*ggas” (52%), “blacklivesmatter” (5%), “Chinese” (4%), white people (3%), and Mexican (2%). There were 2,564,082 tweets about Blacks, 306,012 about Asians, 285,920 about Whites, and 221,281 about Latinx. 

### 3.1. Quantitative Sentiment Analysis of Tweets Using Race-Related Keywords

**Figure 1:**
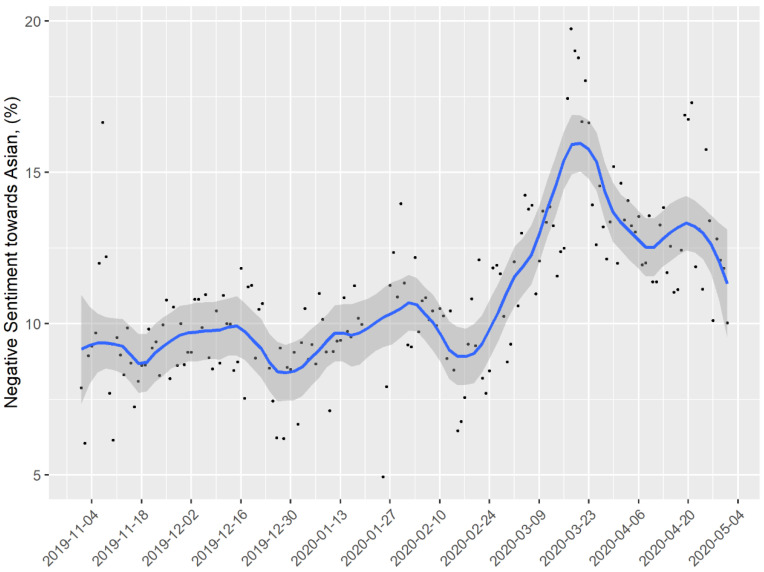
Temporal changes in negative Asian sentiment, November 2019–April 2020. n235,263 tweets. LOESS (locally estimated scatterplot smoothing) was performed to fit smooth curves to the daily prevalence of negative sentiment. The shaded area represents 95% confidence bands around the smoothed trend line.

The proportion of tweets referencing Asians that were negative increased from 9.79% at the beginning of November 2019 to 16.49% at the end of March 2020, a 68.4% increase (Figure 1). At the end of April 2020, the proportion of tweets referencing Asians that were negative declined to 13.2% (Figure 1), still higher than in November 2019. By the end of our follow-up, in June 2020, anti-Asian sentiment had declined but remained substantially elevated compared to levels in 2019 prior to the emergence of the novel coronavirus. In contrast, the proportion of negative tweets referencing other racial and ethnic minorities was substantially higher than tweets specifically referencing Asians but remained relatively stable during this time period, declining less than 1% for tweets referencing Blacks from 48.34% in November to 48.18% in April and increasing by 2% for tweets referencing Latinx (12.81% in November to 13.11% in April), (Table 1). In examining the temporal plots, tweets referencing Blacks had the highest proportion of negative tweets of all racial/ethnic groups but changed little over the same time period from 48.9% in beginning of November 2019 to 48.1% in the end of April 2020 (online supplemental Figure S1). Temporal changes in negative sentiment for tweets referencing Latinx also showed a stable pattern (online Supplemental Figure S2). 

In sensitivity analyses, we examined negative sentiment of tweets using the following terms more specific to Chinese people: “Chinese,” “chinaman,” “ching chong,” and “chink.” The average sentiment of tweets using Chinese-related keywords closely follows sentiment for tweets using Asian related terms in general (online supplemental Table S1). For example, in March 2020, the proportion of tweets using Asian-related keywords that were negative was 15.2% compared to 15.7% of tweets using Chinese specific keywords. In April, 13.1% of tweets using Asian-related keywords were negative compared to 13.2% of tweets using Chinese-related keywords. These results indicate that antipathy towards Chinese people had spillover effects to Asians in general.

The proportion of tweets referencing Asians that were positive declined beginning in February to April (Table 2). For Blacks, Latinx, and Whites, positive sentiment trends remained steady during this time period. Temporal trends of positive and negative Asian sentiment by U.S. states are presented in the online supplemental Figures S3 and S4 and show that for many states, a pattern of rising negative sentiment and declining positive sentiment from February to April.

### 3.2. COVID-19 and Race-Related Tweets

Of the tweets using race-related terms from February to April 2020, 61,228 tweets referenced COVID-19. The top COVID-19 related terms representing 95% of tweets were: virus, covid, Chinese virus, quarantine, rona, pandemic, wuhan, xenophobia, plague, social distancing, epidemic, stay at home, and ncov (Table 3). 

### 3.3. Qualitative Content Analysis: Themes

**Figure 2:**
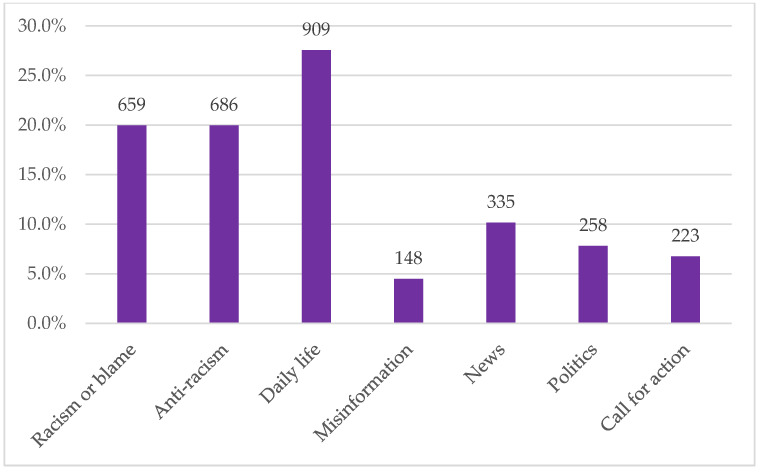
Percent and number of tweets in each content analysis category.

In the random sample of 3,300 tweets for the thematic analysis, 20% had themes expressing racism or blame, 20% had themes expressing anti-racism, and 27% tweets related to daily life impacted by the pandemic. Less common themes included tweets about misinformation (4%), news (10%), politics (8%), and call for action (7%) (Figure 2). Tweets that mentioned President Trump accounted for 18% of tweets across the categories. This qualitative content analysis focuses on understanding the meaning of the tweets, so we provide a detailed description of each theme, temporal patterns from February to April. Illustrative example tweets are presented in Table 4.

### 3.4. Racism & Blame

In February and March 2020, the phrase “Chinese virus” or variants of this were frequently debated on Twitter (Table 4). Many tweets blamed the Chinese government for mishandling the emergence of the pandemic and for providing insufficient or misleading information. Other Twitter users blamed the U.S. government for the handling of the COVID-19 pandemic. Tweets expressing racism and blame towards Asians for the pandemic were commonly interwoven. Some of the tweets also referenced conspiracy theories related to a weaponized virus or the intentional leaking of the virus for financial gain. Other tweets focused not on the government, but on Chinese people for behaviors that led to the emergence of the virus. These included tweets saying that Chinese people ate bats or other animals, which led to cross-species transmission. This locating of blame to the Chinese government or Chinese people was the primary justification for racist rhetoric that included not only prejudicial language, but calls to bomb China or attack Chinese people. These negative sentiments also spilled over into antipathy towards other Asian groups as well (e.g., Vietnamese, Koreans). This spillover is likely due to the “Asians all look alike” phenomenon documented in the literature, which suggests that many non-Asians conflate the various Asian groups [32]. In April, some tweets indirectly expressed support for the term “Chinese virus” or its variants by using it to comment on the virus, political ramifications, or urge certain actions to be taken such as boycotting Asian businesses and products. Tweets expressing racism also employed long-standing racial and ethnic stereotypes, such as ideas about immigrants or foreigners to the U.S. harboring disease. 

### 3.5. Anti-Racism

Many tweets argued that racism was exacerbating the negative effects of the pandemic. In March, some Twitter users were critical of President Trump for publicly using the term “Chinese virus.” Some Asian American Twitter users shared their personal experiences with harassment and discrimination during the pandemic. In February and March, there was a focus on the condemnation of attaching a nationality to the virus and speaking out against racism towards Asians. In April, there was greater focus on the condemnation of racism more broadly. Some Twitter users stated that there was racial bias in the enforcement of social distancing policies in parks or public spaces based on the race/ethnicity of the community member. 

### 3.6. Misinformation 

A subset of tweets contained misinformation along several categories. These included misinformation about the susceptibility to COVID-19 infection of various racial and ethnic groups. In February and March, some users stated there was an increased susceptibility for Whites and Asians and less susceptibility for Blacks. Some tweets also expressed the belief that COVID-19 was created in a Chinese lab (i.e., “The Chinese Communist run Bioweapons research lab created this super viral weapon.”) Misinformation also included unsupported statements about treatment or preventive measures. 

### 3.7. News 

Tweets related to news discussed a variety of domestic and international current events including, cases of COVID-19 in different geographic regions, economic impact of the pandemic, domestic and international response to COVID-19, and information about the virus, treatment, and preventive measures. In April, there was an increased discussion of higher infection and mortality rates among Blacks, indigenous groups, and other racial and ethnic minoritized populations with structural racism as a driver of these health disparities.

### 3.8. Politics

Tweets about politics often referenced Trump and the upcoming presidential election. Some tweets supported President Trump, his response to the pandemic, and his use of the term “Chinese virus.” Others criticized Trump and his response to the pandemic. Some Twitter users stated that President Trump is attempting to shift blame. Commonly, tweets were polarizing and displayed negativity towards either Democrats or Republicans.

### 3.9. Call to Action

Some Twitter users emphasized the importance of preventive measures including hand washing, social distancing, and mask wearing in February and March. Others expressed the need to provide increased funding for COVID-19 efforts and to support small businesses through patronage. In April, there was also discussion of racial and ethnic disparities in infection, morbidity, and mortality from COVID-19 especially among African Americans and the need to address these disparities impacting minority, immigrant, and prisoner populations. 

### 3.10. Daily Life

Tweets about daily life described disruptions to plans and people’s experiences sheltering in place. Some of these tweets touched upon the impact of the pandemic on relationships, the development of new hobbies, and the cancellation of events such as in-person graduation ceremonies and travel. A few tweets centered around people not following social distancing measures. 

## 4. Discussion

Our mixed-methods study of over 3.4 million tweets shows a marked increase in anti-Asian sentiment and a decline in positive Asian sentiment following the emergence of COVID-19 in the U.S. Sensitivity analyses using Chinese specific keywords showed a similar temporal pattern to overall negative Asian sentiment, indicating antipathy towards Chinese people had spillover effects to Asians in general. 

Google searches for “Wuhan Virus” and “Chinese Virus” spiked in late January (online supplemental Figure S5). The WHO released guidelines in February 2020 cautioning against the use of stigmatizing language in reference to COVID-19 [33]. However, in early to mid-March, U.S. politicians including Republican Paul Gosar, Mike Pompeo, and President Trump used the terms “Wuhan virus” or “Chinese virus” [34]. Google searches for these terms again spiked and retweets using “Chinese virus” or related terms increased by 650% on 8 March. News media articles using these terms increased by 800% on 9 March [33]. 

This study finding aligns with reports from the FBI, newspapers, and other commentaries about a rise in anti-Asian hate crimes during the same period [3]. Our results are also consistent with a recent study using Project Implicit data and finding that Implicit Americanness Bias—or the subconscious belief that European American individuals are more “American” than Asian American individuals—declined steadily from 2007 through early 2020 but reversed trend and began to increase on 8 March, following the increase in stigmatizing language related to COVID-19 [35].

Our content analyses of the tweets enriched the quantitative analysis by identifying several interrelated themes related to racist rhetoric, conspiracy theories, misinformation and blame. It is noteworthy that negative sentiment existed prior to the emergence of COVID-19, and was generally higher for other racial and ethnic groups (e.g., African Americans and Latinx) compared with Asians. However, an important observation was that the increase in negative tweets occurred only for Asians between February to April, corresponding to the emergence of concern and social distancing practices in the U.S. Moreover, the apex was the week of March 16, corresponding to the controversy surrounding President Trump’s decision to use the phrase, “Chinese virus.” The World Health Organization has cautioned against using such a phrase to avoid stigma [33]. The temporal patterns seen herein provide further evidence of a specific effect of the pandemic on anti-Asian sentiment, and not just a general rise in racial tensions. 

Just as importantly, our qualitative analysis documented anti-racist sentiment. Many tweets condemned racist rhetoric, expressed solidarity towards the Asian community and sympathy for hate crime victims. This highlights the key role that counter-messaging can play in dampening negative speech online, as well as the potential to garner both social support for individuals, and social movements to support entire communities.

This study has important limitations. Twitter data represent what people are willing to express in the online public sphere. These may differ from in-person interactions and discussions. However, the sense of anonymity online can permit some people to express views they would not express in in-person interactions or endorse in traditional surveys. Twitter can suspend users who tweet abusive or threatening content [36], and these users could have been suspended before our data were collected. Twitter users are not representative of the U.S. population. Compared to the general adult population in the U.S., adult Twitter users are younger and more educated [37]. For example, 29% of adult Twitter users are between 18–29 years of age, and this age group makes up 21% of all U.S. adults. Forty-two percent of Twitter users have a college degree compared to 31% of the adult U.S. population. Twitter users also are slightly more racially/ethnically diverse than the overall adult U.S. population. Among adult Twitter users, 11%, 17%, and 64% are Black, Hispanic, and White, respectively compared to 11%, 15%, and 60% of U.S. adults. Twitter users are similar to U.S. adult population in terms of gender with 52% of adult Twitter users being female [37]. 

Furthermore, our data are bound to the time periods studied and do not capture the important events that have arisen recently in response to the death of African Americans by police officers and the ensuing protests. Those analyses may be the topic of a future study, however. In addition, our analyses were limited to English-language tweets, which may lead to certain biases (e.g., underestimation or overestimation of racist tweets). Finally, our analyses should not be seen as providing causal evidence that COVID-19 resulted in anti-Asian bias, but rather, as documenting that the two phenomena are interrelated. 

There are a number of directions for future investigation. An essential question is whether the racial sentiments expressed on Twitter translate to racist actions in other venues and how long these attitudes persist. In our follow-up, we saw a rapid decline in negative sentiment within weeks of the peak, but the percent of negative tweets had not yet returned to pre-virus levels within the 3 months of our follow-up period. Future work is needed to examine longer-term temporal changes in racial attitudes in response to events. 

Future work might examine geographical variation in anti-Asian bias and possible predictors, including variation in area-level COVID-19 infection and mortality rate, demographics such as the density of Asians versus other racial and ethnic groups, economic disadvantage, political affiliation, among other factors. Additionally, future work might investigate how anti-Asian bias may have affected economic and health outcomes of Asians during the pandemic, given reports of economic hardships faced by Asian businesses and experiences with discrimination and harassment that may have an impact on mental health and physical health outcomes influenced by stress and anxiety. Previous research shows that both short-term acute stress experiences as well as prolonged stress, particularly due to social identity threat, has lasting physiologic impacts [38,39]. Further, even when not the target of direct racial discrimination or harassment, vicarious racial discrimination has been associated with poorer mental and physical health [40], especially among those with higher levels of racial group identification [41].

Despite these limitations, our study provides empirical evidence to support the numerous anecdotal reports of anti-Asian bias that have surfaced in the recent media. This study adds to the literature in several ways. We provide new empirical evidence that there may be effects of COVID-19 on a specific racial group that does not appear to affect other racial groups (e.g., African Americans). This suggests that interventions should not only address power, equity, and inclusion in a global fashion for all groups, but also address group-specific issues. Additionally, our qualitative findings provide previously unreported themes that could be studied in future research. For example, to what extent do anti-racism messages counterbalance hateful messages and how long do these increases in negative racial sentiment persist? 

## 5. Conclusions

Our study gains strength from a mixed-methods approach that employs both sentiment and content analyses that involves a transdisciplinary collaboration of computer scientists, public health experts, and qualitative researchers. Our analyses imply that public health threats, such as a viral pandemic, can have spillover effects on race relations. Our findings join a broader corpus of research that has shown that racial discrimination is associated with health problems. Thus, future responses to health crises must not only address medical outcomes, but social outcomes as well. Finally, prior data suggests that public sentiment about the nature of disparities (victim blaming vs. discrimination) corresponds to sentiment about the provision of shared resources and social policies and programs to support those very groups [14,42]. Hence, the awareness of negative racial attitude may have policy implications that are critical for the health and well-being of stigmatized racial groups. 

## Figures and Tables

**Table 1:** Percent of tweets referencing racial and ethnic groups that are negative by month, November 2019–June 2020.

	November	December	January	February	March	April	May	June
Racial/ethnic group	% (*n*)	% (*n*)	% (*n*)	% (*n*)	% (*n*)	% (*n*)	% (*n*)	% (*n*)
Asian	9.45 (31,774)	9.34 (30,476)	9.96 (27,588)	9.81 (32,925)	15.21 (65,915)	13.11 (46,585)	12.02 (40,356)	13.41 (30,150)
Black	48.34 (298,548)	48.22 (321,531)	48.37 (253,019)	45.15 (280,354)	47.63 (286,452)	48.18 (275,009)	46.77 (342,085)	38.15 (504,600)
Latinx	12.81 (27,466)	13.14 (26,831)	13.15 (21,401)	12.51 (33,258)	12.02 (28,310)	13.11 (24,385)	15.58 (27,981)	21.63 (31,357)
White	44.96 (27,496)	46.14 (30,019)	46.92 (23,511)	45.80 (27,749)	46.36 (27,240)	46.01 (25,960)	52.57 (51,425)	50.57 (72,161)

Percentages refer to percent of tweets in each racial/ethnic category that are negative. *n* refer to the total number of tweets for that racial/ethnic category for that month.

**Table 2:** Percent of tweets referencing racial and ethnic groups that are positive by month, November 2019–June 2020.

	November	December	January	February	March	April	May	June
Racial/ethnic group	% (*n*)	% (*n*)	% (*n*)	% (*n*)	% (*n*)	% (*n*)	% (*n*)	% (*n*)
Asian	15.10 (31,774)	15.95 (30,476)	16.90 (27,588)	15.20 (32,925)	8.63 (65,915)	11.30 (46,585)	13.01 (40,356)	12.07 (30,150)
Black	4.37 (298,548)	4.47 (321,531)	4.48 (253,019)	5.90 (280,354)	4.28 (286,452)	4.14 (275,009)	4.43 (342,085)	6.56 (504,600)
Latinx	16.62 (27,466)	16.80 (26,831)	16.84 (21,401)	19.22 (33,258)	15.11 (28,310)	15.88 (24,385)	15.47 (27,981)	12.16 (31,357)
White	3.66 (27,496)	3.63 (30,019)	3.69 (23,511)	3.64 (27,749)	3.59 (27,240)	3.57 (25,960)	52.57 (51,425)	50.57 (72,161)

Percentages refer to percent of tweets in each racial/ethnic category that are negative. *n* refer to the total number of tweets for that racial/ethnic category for that month.

**Table 3:** Top COVID-19 related terms invoked in Tweets mentioning a race-related term, February–April 2020.

Term	*n*	Percent
virus	13,167	21.55
covid	12,347	20.21
chinese virus	8181	13.39
quarantine	6771	11.08
rona	6579	10.77
pandemic	4285	7.01
wuhan	2494	4.08
xenophobia	1548	2.53
plague	936	1.53
social distancing	809	1.32
epidemic	680	1.11
stay at home	387	0.63
ncov	344	0.56
stayhome	338	0.55
coro	268	0.44
curfew	265	0.43
socialdistancing	179	0.29
kung flu	171	0.28
wash your hands	168	0.28
6 feet	147	0.24

Data source: 61,089 tweets from the United States were collected between February 2019 and April 2020 included at least one COVID-19- and one race-related term. From a list of 75 COVID-19 terms, 20 terms comprised 98% of all tweets.

**Table 4:** Content analysis of themes with illustrative examples.

Themes	Example Tweets
Racism	Seeing Chinese people w these mask acting like we the ones that have the virus out here
	Americans are starving and waiting in food lines for a bag of crap. Americans want to work. Not have immigrants stealing jobs
	Border wall to keep illegals out should continue because of the virus
Blame	Thank you China for unleashing this Chinese virus into the world.
	The present crisis is a result of Trump’s ineptitude and inaction.
	People being racist towards Chinese people because of the virus is like saying I got the flu cuz I’m white. I guess ethnicity now plays a part in the forces of nature? Being a racist isn’t going to solve the problem.
Anti-racism	The real virus is the racism and hate that is spread from one generation to another. Let’s do better. Let’s be better.
	You cannot be serious. Do not call it the Chinese Virus. You’re a racist idiot if you do
Misinformation	...Chinese Communist run research lab created this super viral weapon
	On the Orange line this morning...man was telling his friend that he’s “not gonna get corona and neither are you” because “only White and Asian people get it.”
News	Misguided virus fears said to be hitting Asian American businesses
	Most Louisiana Carona deaths are in New Orleans where blacks make up 60% of the population and many are in the service industry.
Politics	He knows it is going to get worse a lot worse. He’s setting up the scapegoat so he can flame xenophobia & shift blame before the election. It isn’t just ignorant racism, it’s a calculated political maneuver…
Call to action	Good Night World! Please Stay Safe & Healthy. StayAtHome SocialDistance6Ft ThisWillPass TemporaryNormal BeatItCorona PeaceAndLove AllLivesMatter
	As of today, all COVID19 deaths in City of St. Louis are African Americans. This reinforces the health disparities that existed before this virus, but also compels us to action now.
Daily Life Impacted by COVID-19	So a lot of side n*ggas about be lonely during this pandemic guess u about see how much of a side n*gga u are.
	At this point in quarantine, a n*gga just want a hug
	I had graduation planned fa 14 years u think carona cared no…

Some tweets were edited or shortened to remove identifying information. Hashtags, urls, and tags were removed. Data source: 61,089 tweets from the United States were collected between February 2019 to April 2020 included at least one COVID-19 and race-related term.

## Supplementary Materials

The following are available online at https://www.mdpi.com/1660-4601/17/19/7032/s1, Figure S1: Temporal changes in negative Black sentiment, November 2019–April 2020, Figure S2: Temporal changes in negative Latinx sentiment, November 2019–April 2020, Figure S3: Temporal changes in negative Asian sentiment by state, Figure S4: Temporal changes in positive Asian sentiment by state, Figure S5: Temporal trends in Google Searches of “wuhan virus” and “china virus”, Table S1: Percent negative tweets using Asian and Chinese-related keywords by month, November 2019–June 2020.
