# Supplementary material for: Exploring U.S. Shifts in Anti-Asian Sentiment with the Emergence of COVID-19
Source: Int J Environ Res Public Health. 2020 Sep 25;17(19):7032. doi: 10.3390/ijerph17197032 (PMC7579565; doi:10.3390/ijerph17197032)
Supplement: Supplementary file 1 [file ijerph-17-07032-s001.pdf]

Online Supplementary Materials

Figures S1 – S5, Tables S1

Figure S1. Temporal changes in negative Black sentiment, November 2019 – April 2020

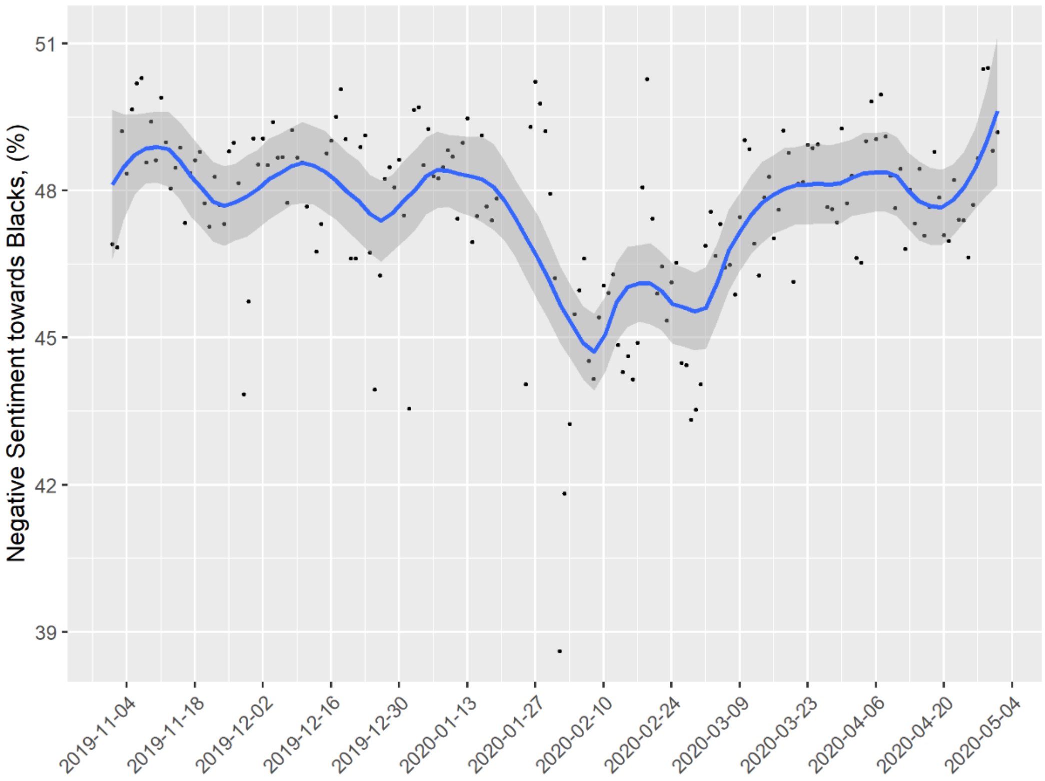

N=1,714,913 tweets. LOESS (locally estimated scatterplot smoothing) was performed to fit smooth curves to daily prevalence of negative sentiment. The shaded area represents 95% confidence bands around the smoothed trend line.

Figure S2. Temporal changes in negative Latinx sentiment, November 2019 – April 2020

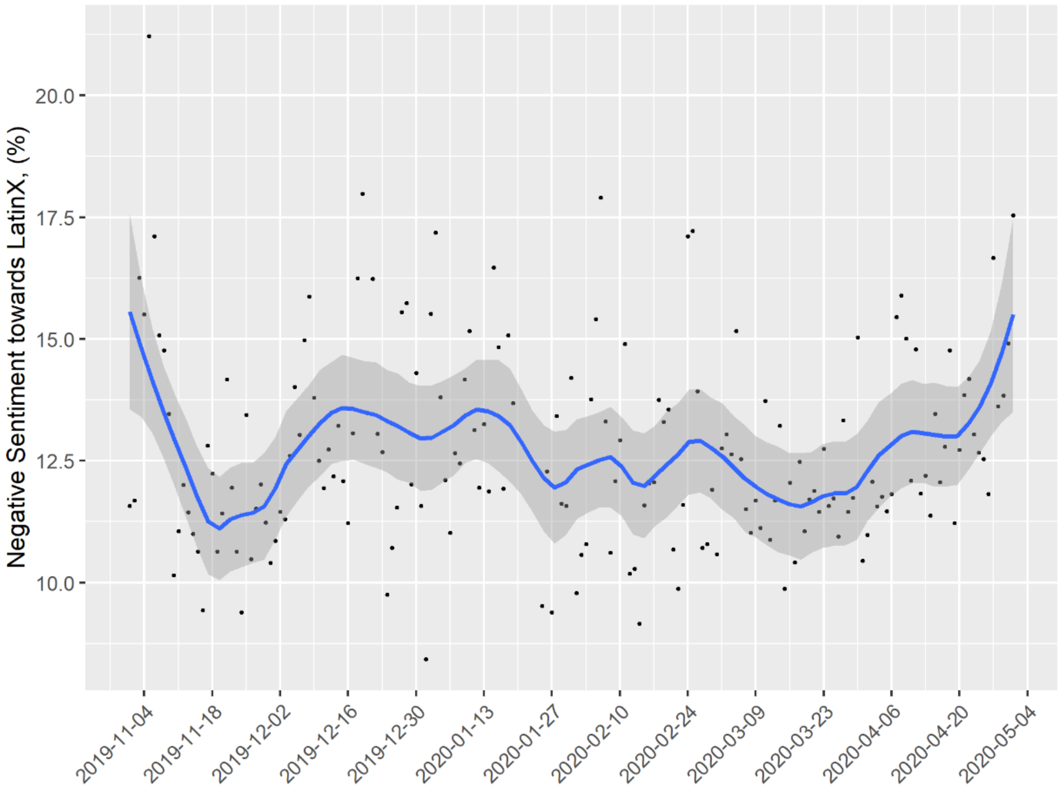

N=161,651 tweets. LOESS (locally estimated scatterplot smoothing) was performed to fit smooth curves to daily prevalence of negative sentiment. The shaded area represents 95% confidence bands around the smoothed trend line.

Figure S3. Temporal changes in negative Asian sentiment by state

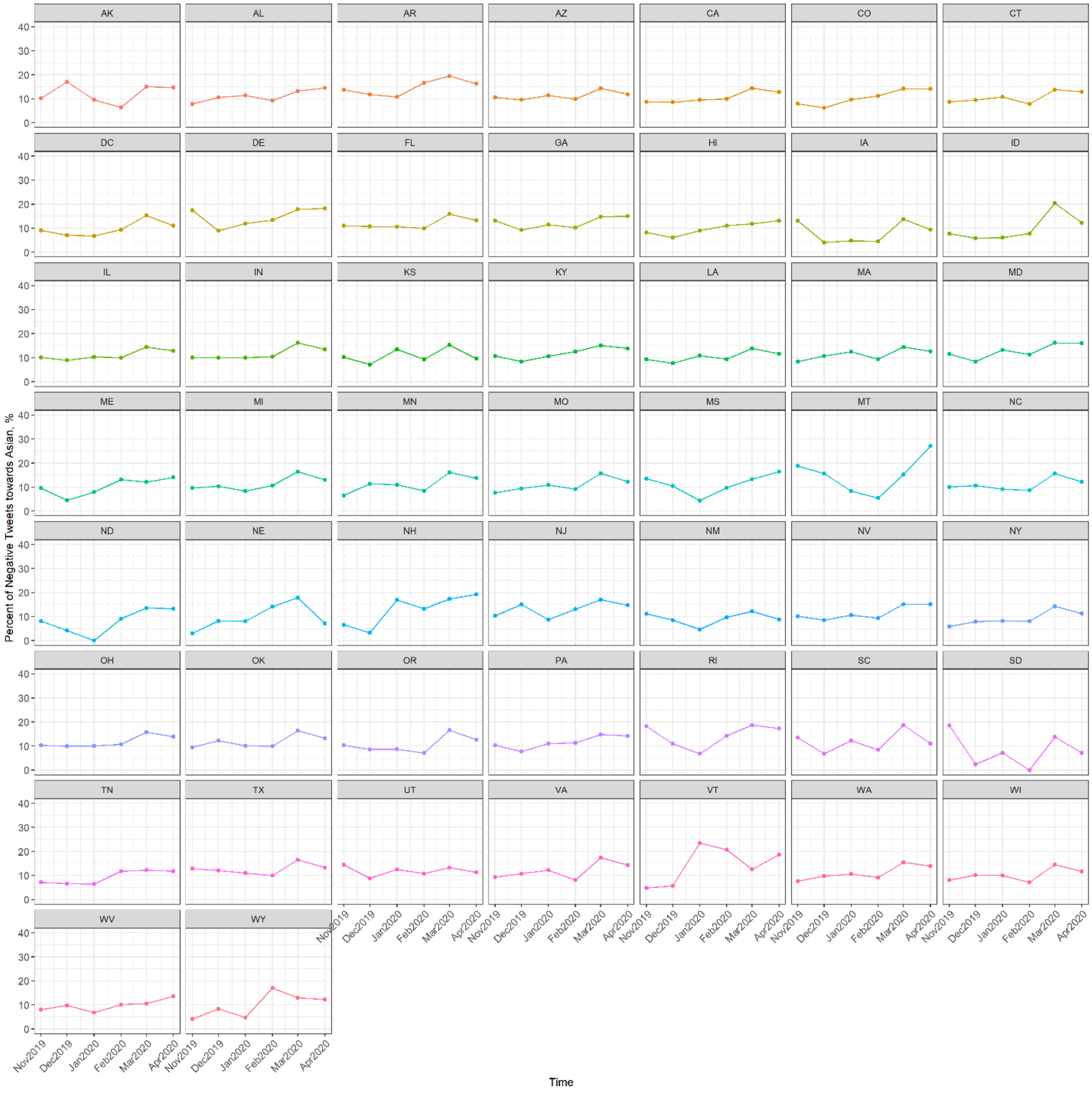

Figure S4. Temporal changes in positive Asian sentiment by state

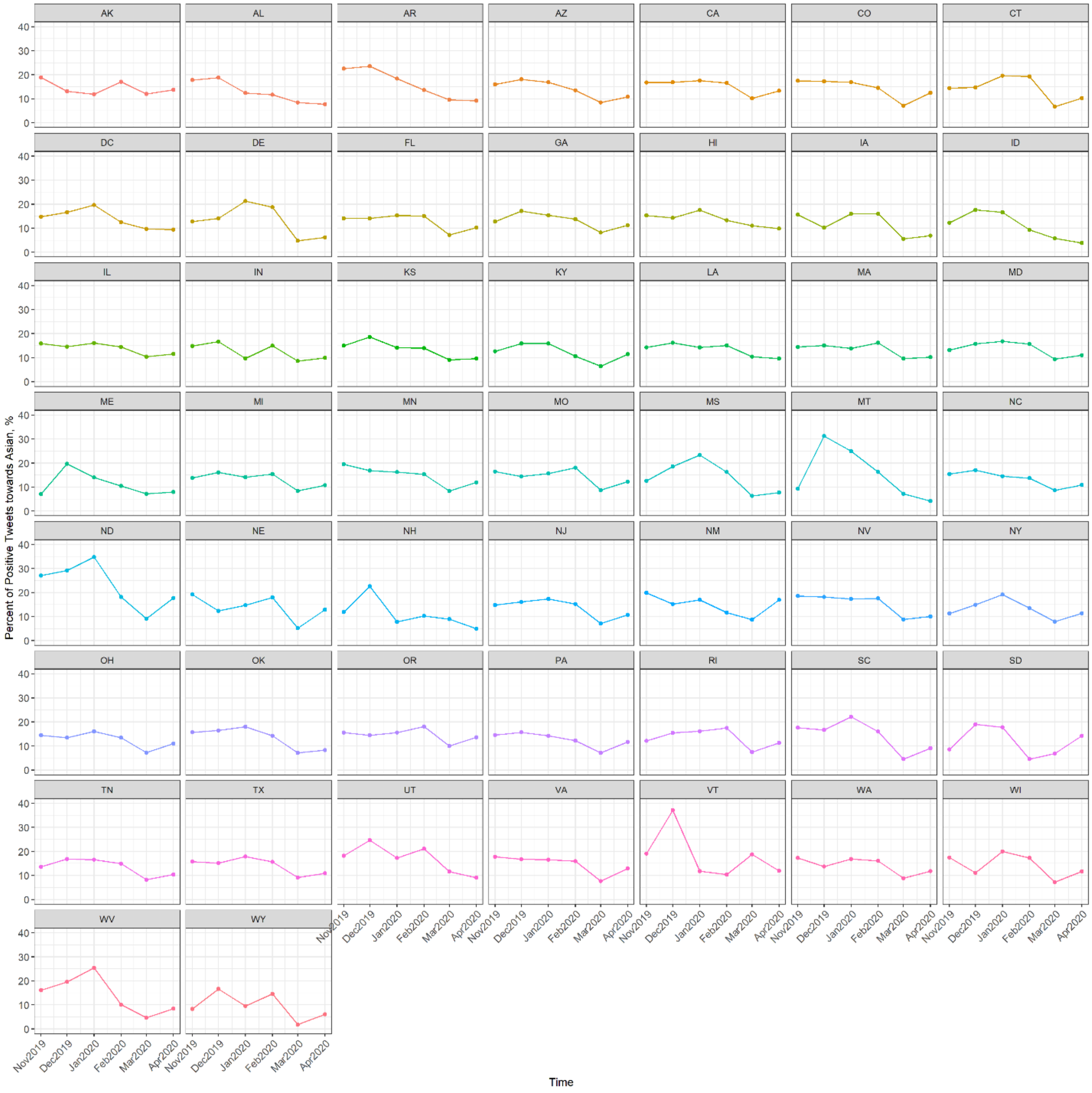

Figure S5. Temporal trends in Google Searches of “wuhan virus” and “china virus”

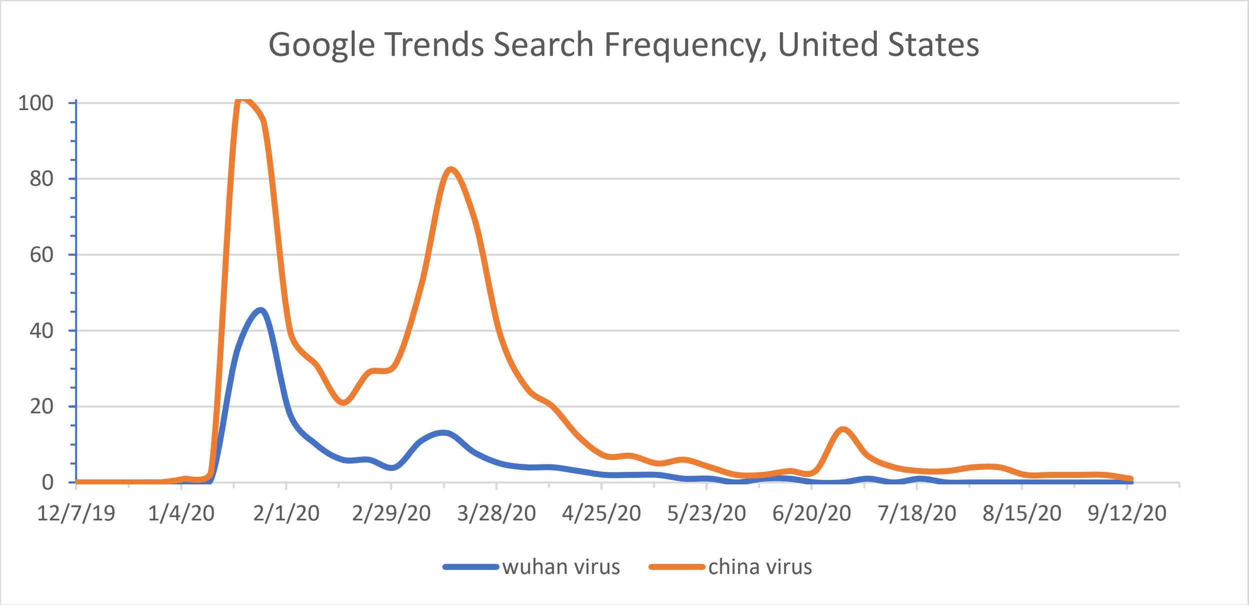

Table S1. Percent negative tweets using Asian and Chinese-related keywords by month, November 2019-June 2020.

	**November**	**December**	**January**	**February**	**March**	**April**	**May**	**June**
Racial/ethnic group	% (N)	% (N)	% (N)	% (N)	% (N)	% (N)	% (N)	% (N)
Asian	9.45 (31,774)	9.34 (30,476)	9.96 (27,588)	9.81 (32,925)	15.21 (65,915)	13.11 (46,585)	12.02 (40,356)	13.41 (30,150)
Chinese	5.82 (9,558)	6.78 (9,277)	8.20 (8,857)	8.89 (10,834)	15.75 (37,780)	13.17 (23,279)	11.42 (14,952)	11.11 (9,797)

Percentages refer to percent of tweets in each racial/ethnic category that are negative. N refer to the total number of tweets for that racial/ethnic category for that month.
